# The Rise of NK Cell Checkpoints as Promising Therapeutic Targets in Cancer Immunotherapy

**DOI:** 10.3389/fimmu.2019.02354

**Published:** 2019-10-17

**Authors:** Haoyu Sun, Cheng Sun

**Affiliations:** ^1^The First Affiliated Hospital of USTC, Division of Life Sciences and Medicine, University of Science and Technology of China, Hefei, China; ^2^Division of Molecular Medicine, Hefei National Laboratory for Physical Sciences at Microscale, The CAS Key Laboratory of Innate Immunity and Chronic Disease, School of Life Sciences, University of Science and Technology of China, Hefei, China; ^3^Institute of Immunology, University of Science and Technology of China, Hefei, China

**Keywords:** NK cell, NK cell exhaustion, inhibitory receptor, checkpoint inhibitor, cancer immunotherapy

## Abstract

Checkpoint immunotherapy that targets inhibitory receptors of T cells, thereby reversing the functional exhaustion of T cells, marks a breakthrough in anticancer therapy. The success of T cell-directed checkpoint inhibitors of CTLA-4 and PD-1/PD-L1 has opened a new approach for cancer immunotherapy and resulted in extensive research on immune checkpoints. However, it is only in recent years that research on NK cell exhaustion and potential checkpoints impacting NK cells has become popular. NK cells, as the major player in innate immunity, are critical for immune surveillance, particularly the control of metastasis and hematological cancers. The balance between activating and inhibitory signals fine tunes the activation and effector functions of NK cells, and transformed cells modulate NK cells by upregulating negative signaling that “exhausts” NK cells. Exhausted NK cells with excessive expression of inhibitory receptors (checkpoint molecules) are impaired in the recognition of tumor cells as well as antitumor cytotoxicity and cytokine secretion. Therefore, an understanding of the potential checkpoint molecules involved in NK cell exhaustion is particularly important in terms of NK cell-targeted cancer immunotherapy. In this review, we summarize recent advances in NK cell checkpoint inhibitors and their progress in clinical trials. Moreover, we highlight some of the latest findings in fundamental NK cell receptor biology and propose potential NK cell checkpoint molecules for future immunotherapeutic applications.

## Introduction

Excessive negative regulation of immune cells by inhibitory receptors (checkpoint molecules) results in functional exhaustion of these cells, which is one of the major reasons for tumor escape. The activation and function of immune cells are regulated through activating and inhibitory receptors on these cells, and establishing an equilibrium between activating and inhibitory signaling is critical because it assures effective control against pathogenic factors (such as tumors, viruses, and bacteria) meanwhile helping to avoid self-directed attacks (such as autoimmune disease) (1, 2). The negative feedback provided by inhibitory receptors is the key to immune regulation; however, unfortunately, tumor cells can take advantage of this negative feedback system, as they upregulate the surface expression of corresponding ligands to ingratiate excessive expression of inhibitory receptors on immune cells that automatically leads to reduced activation and functional exhaustion of these cells (3, 4). Antibodies that specifically target these inhibitory checkpoints can effectively block the interaction between the checkpoint molecule and its ligands, thereby reversing the functional exhaustion of immune cells and restoring antitumor immunity.

Checkpoint immunotherapy targeting checkpoint molecules that reverses functional exhaustion in immune cells marks a major breakthrough in anticancer therapy. Blocking inhibitory receptors on T cells to reverse functional exhaustion in these cells has made great progress. T cells from cancer patients highly express inhibitory receptors including cytotoxicity T-lymphocyte-associated protein 4 (CTLA-4), programmed cell death protein 1 (PD-1), T cell immunoglobulin- and mucin-domain-containing molecule 3 (TIM-3), lymphocyte activation gene 3 (LAG-3), T cell immunoreceptor with Ig and ITIM domains (TIGIT), etc., which contribute to T cell functional exhaustion (5, 6). Blocking these checkpoint molecules can effectively reverse T cell exhaustion and restore the antitumor capacity of T cells. During the past 10 years, the efficacy and feasibility of checkpoint immunotherapy have been verified in clinical settings, and antibodies targeting CTLA-4, PD-1/programmed death-ligand 1 (PD-L1), TIM-3, LAG-3, or TIGIT have entered clinical trials (7, 8). Monoclonal antibodies (mAbs) targeting CTLA-4 and PD-1/PD-L1 were approved by the U.S. Food and Drug Administration (FDA) in 2011 and 2014, respectively. The combined use of an anti-CTLA-4 mAb with an anti-PD-1 mAb showed better efficacy than either antibody used as a monotherapy (9–11).

However, although remarkable clinical benefits derived from anti-CTLA-4 and anti-PD-1/PD-L1 antibody therapies have been noted in some patients, there are still many patients who do not respond to these treatments. Currently, researchers are still trying to understand these “non-responders;” additional unknown inhibitory pathways that suppress immune responses could be an explanation for nonresponse. On the other hand, tumors escape T cell-mediated immunity by downregulating the expression of major histocompatibility complex class I (MHC-I) molecules; these MHC-I-null tumor cells are not attacked by T cells, but they are still targets of natural killer (NK) cells (12). Moreover, Zhang et al. demonstrated the significance of NK cells in cancer immunotherapy and noted that mAb targeting checkpoint molecule TIGIT, which is expressed by both T cells and NK cells, could improve the antitumor immunity of both T and NK cells. Furthermore, they found that TIGIT blockade was effective even in the absence of T cells and B cells, highlighting the importance of NK cells in checkpoint-targeted immunotherapy (13, 14).

**Figure 1:**
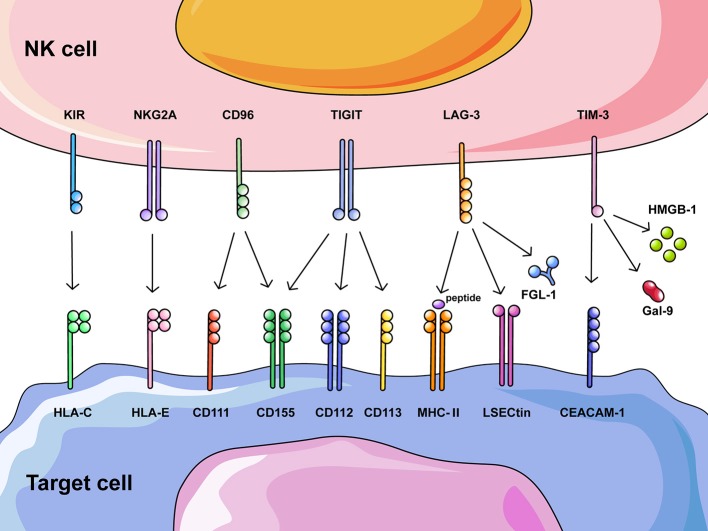
Overview of potential NK cell checkpoint molecules and their corresponding ligands. Recognition and clearance of tumor cells by NK cells are regulated through activating and inhibitory receptors on NK cells that bind their corresponding ligands on tumor cells. Increased expression of ligands on tumor cells induces altered expression of inhibitory receptors on NK cells, excessive negative regulation results in functional exhaustion of NK cells. This figure summarizes inhibitory receptors on NK cells that could also act as checkpoints in cancer immunotherapy, including HLA class I-specific receptors (KIR and NKG2A) and those recognizing ligands other than HLA class I molecules (CD96, TIGIT, LAG-3, and TIM-3).

NK cells are innate lymphocytes that play a critical role in the early defense against transformed cells, and they are particularly important in the control of cancer metastasis and hematological cancers (1, 15–17). NK cells can directly kill tumor cells, secrete various cytokines such as interferon (IFN)-γ and tumor necrosis factor (TNF)-α to initiate antitumor responses, and recruit other immune cells into the antitumor response (1, 18, 19). Alterations in NK cells, for example, excessive expression of inhibitory receptors or reduced expression of activating receptors, can result in impaired cytotoxicity against tumor cells and a decreased ability to recruit other immune cells (20–22). Some checkpoint molecules in cytotoxic T lymphocytes (CTLs), such as PD-1, LAG-3, TIGIT, and TIM-3, are shared with NK cells. Blockade with checkpoint inhibitors that reverses the functional exhaustion of NK cells opens a new strategy for cancer immunotherapy that may complement the limitations of T cell-based immunotherapy. This review summarizes recent advances in NK cell checkpoint molecules in humans (Figure 1) and the corresponding antibodies being studied in clinical trials (Tables 1, 2). In addition, we highlight some of the latest findings in fundamental NK cell receptor biology that may provide a fundamental basis for future NK cell-based immunotherapeutic applications.

**Table 1:** Potential NK cell checkpoint molecules in cancer.

**Targets**	**Monoclonal antibody**	**Expression distribution**	**Ligand**	**Signaling motif**
**KILLER CELL LECTIN-LIKE RECEPTOR FAMILY**				
NKG2A	Monalizumab, IPH2201	CD8^+^ T cells and NK cells	HLA-E	ITIM
**KILLER CELL IMMUNOGLOBULIN-LIKE RECEPTOR FAMILY**				
KIR	IPH2101, 1-7F9, Lirilumab, and IPH4102	CD4^+^, CD8^+^ T cells, NK cells	MHC class I molecules	ITIM/ITAM
**IMMUNOGLOBULIN SUPERFAMILY**				
TIGIT	MTIG7192A, OMP-313M32, and AB154	CD4^+^, CD8^+^ T cells, NK cells	CD155, CD112, CD113	ITIM/ITT
CD96	–	CD4^+^, CD8^+^ T cells, NK cells	CD155	ITIM/YXXM
LAG-3	Sym022, BMS-986016, Relatlimab, IMP321, and Eftilagimod Alpha	CD4^+^, CD8^+^ T cells, NK cells, B cells, and dendritic cells	MHC class II molecules, Fibrinogen-like Protein 1	KIEELE
TIM-3	sym 023, TSR-022, LY3321367, BGB-A425, and MBG453	CD4^+^, CD8^+^ T cells, dendritic cells, NK cells, and monocytes	Gal-9, phosphatidylserine, HMGB1, Ceacam-1	Tyrosine

**Table 2:** Clinical trials based on potential NK cell checkpoint inhibitors in cancer.

	**Registry**	**Disease**	**Intervention**	**Phase**	**Status**	**Enrollment**	**Sponsors and collaborators**
TIGIT	NCT03119428	Locally advanced cancer, metastatic cancer	OMP-313M32/Nivolumab	I	Active	30	OncoMed Pharmaceuticals, Inc.
	NCT03563716	Non-small cell lung cancer	MTIG7192A/Atezolizumab	II	Active	120	Genentech, Inc.
	NCT03628677	Non-small Cell Lung Cancer, squamous cell carcinoma of the head and neck, renal cell carcinoma, breast cancer, colorectal cancer, melanoma, bladder cancer, ovarian cancer, endometrial cancer, merkel cell carcinoma, gastroesophageal cancer	AB154/AB122	I	Recruiting	242	Arcus Biosciences, Inc.
	NCT02794571	Advanced/metastatic tumors	Atezolizumab/MTIG7192A	I	Recruiting	300	Genentech, Inc.
KIR	NCT01750580	CANCER, NOS	Lirilumab/Ipilimumab	I	Completed	22	Bristol-Myers Squibb
	NCT01714739	CANCER, NOS	Lirilumab/Nivolumab/Ipilimumab	I/II	Active	337	Bristol-Myers Squibb
	NCT03203876	Advanced cancer	Lirilumab/Nivolumab/Ipilimumab	I	Active	21	Bristol-Myers Squibb Ono Pharmaceutical Co.Ltd
	NCT01222286	Smoldering multiple myeloma	IPH2101	II	Completed	30	Innate Pharma
	NCT00999830	Multiple myeloma	IPH2101	II	Completed	27	Innate Pharma
	NCT00552396	Multiple myeloma	Anti-KIR (1-7F9)	I	Completed	32	Innate Pharma
	NCT01256073	Acute myeloid leukemia	IPH2101	I	Completed	21	Innate Pharma
	NCT01687387	Acute myeloid leukemia	IPH2102	II	Completed	152	Innate Pharma
	NCT02481297	Leukemia, chronic lymphocytic leukemia, lymphocytic leukemia	Lirilumab/Rituximab	II	Active	8	M.D. Anderson Cancer Center ristol-MyersSquibb
	NCT02593045	Cutaneous T-Cell lymphoma	IPH4102	I	Active	60	Innate Pharma
	NCT03902184	Lymphoma, mycosis fungoides/sezary syndrome	IPH4102 + Gemcitabine + Oxaliplatin	II	Recruiting	250	Innate Pharma
TIM-3	NCT03489343	Metastatic cancer, solid tumor, lymphoma	Sym023	I	Recruiting	48	Symphogen A/S
	NCT02817633	Advanced or metastatic solid tumors	TSR-022/TSR-042/TSR-033	I	Recruiting	819	Tesaro, Inc.
	NCT03680508	Adult primary liver cancer, advanced adult primary liver cancer, localized unresectable adult primary liver cancer	TSR-022 + TSR-042	II	Not yet recruiting	42	University of Hawaii
	NCT03311412	Metastatic cancer, solid tumor, lymphoma	Sym021/Sym022/Sym023	I	Recruiting	102	Symphogen A/S
	NCT03099109	Solid tumor	LY3321367/LY3300054	I	Recruiting	196	Eli Lilly and Company
	NCT03744468	Locally advanced or metastatic solid tumors	BGB-A425/tislelizumab	I/II	Recruiting	162	BeiGene
	NCT03961971	Glioblastoma multiforme	MBG453	I	Not yet recruiting	15	Sidney Kimmel Comprehensive Cancer Center at Johns Hopkins NovartisPharmaceuticals
	NCT03066648	Leukemia, myelodysplastic syndromes, preleukemia, bone marrow diseases, hematologic diseases	Decitabine/PDR001/MBG453	I	Recruiting	175	Novartis Pharmaceuticals
	NCT02608268	Advanced malignancies	MBG453/PDR001	I/II	Recruiting	250	Novartis Pharmaceuticals
LAG-3	NCT03489369	Metastatic cancer, solid tumor, lymphoma	Sym022	I	Recruiting	30	Symphogen A/S
	NCT03311412	Metastatic cancer, solid tumor, lymphoma	Sym021/Sym022/Sym023	I	Recruiting	102	Symphogen A/S
	NCT02061761	Hematologic neoplasms	BMS-986016/BMS-936558	I/II	Recruiting	132	Bristol-Myers Squibb
	NCT02966548	Cancer	Relatlimab/Nivolumab	I	Recruiting	45	Bristol-Myers Squibb Ono Pharmaceutical Co.Ltd
	NCT01968109	Neoplasms by site	Relatlimab/Nivolumab/BMS-986213	I/II	Recruiting	2000	Bristol-Myers Squibb
	NCT03459222	Advanced cancer	Relatlimab/Nivolumab/BMS-986205/Ipilimumab	I/II	Recruiting	230	Bristol-Myers Squibb
	NCT02658981	Glioblastoma, gliosarcoma, recurrent brain neoplasm	BMS 986016/Anti-PD-1/Anti-CD137	I	Recruiting	100	Sidney Kimmel Comprehensive Cancer Center at Johns Hopkins National Cancer Institute (NCI) Bristol-MyersSquibb
	NCT03044613	Gastric cancer, esophageal cancer, gastroesophageal cancer	Nivolumab/Relatlimab/Carboplatin/Paclitaxel/Radiation	I	Recruiting	32	Sidney Kimmel Comprehensive Cancer Center at Johns Hopkins Bristol-MyersSquibb
	NCT03623854	Chordoma, locally advanced chordoma, metastatic chordoma, unresectable chordoma	Nivolumab/Relatlimab	II	Recruiting	20	Jonsson Comprehensive Cancer Center National Cancer Institute(NCI)
	NCT03493932	Glioblastoma	Nivolumab/BMS-986016	I	Recruiting	20	National Institute of Neurological Disorders and Stroke (NINDS)
	NCT03743766	Melanoma	Relatlimab/Nivolumab	II	Recruiting	42	John Kirkwood Bristol-MyersSquibb
	NCT00351949	Stage IV renal cell carcinoma	IMP321	I	Completed	24	Immutep S.A, Umanis
	NCT03252938	Solid tumors, peritoneal carcinomatosis	IMP321/Avelumab	I	Recruiting	50	IKF Klinische Krebsforschung GmbH at Krankenhaus Nordwest
	NCT00349934	Metastatic breast cancer	IMP321	I	Completed	33	Immutep S.A, Umanis
	NCT03625323	Non-small cell lung cancer, squamous cell carcinoma of head and neck	Eftilagimod alpha/Pembrolizumab	II	Recruiting	109	Immutep S.A, Merck Sharp & Dohme Corp.
	NCT02614833	Stage IV breast adenocarcinoma	IMP321/Paclitaxel	II	Active	241	Immutep S.A.
	NCT02676869	Stage IV and stage III melanoma	IMP321/Pembrolizumab	I	Active	24	Immutep Australia Pty. Ltd.
NKG2A	NCT02921685	Hematological malignancy	Monalizumab (IPH2201)	I	Recruiting	18	Institut Paoli-Calmettes InnatePharma
	NCT02557516	Chronic lymphocytic leukemia	Monalizumab	I/II	Active	22	Innate Pharma
	NCT02459301	Gynecologic cancer	IPH2201	I	Active	59	Canadian Cancer Trials Group
	NCT02643550	Head and neck neoplasms	Monalizumab/Cetuximab/ Anti-PD-L1	I/II	Recruiting	140	Innate PharmaAstraZeneca
	NCT02671435	Advanced solid tumors	Durvalumab (MEDI4736)/Monalizumab (IPH2201)	I/II	Recruiting	501	MedImmune LLC
	NCT03822351	Unresectable stage III non-small cell lung cancer	Durvalumab/Monalizumab/ Oleclumab	II	Recruiting	300	MedImmune LLC
	NCT03833440	Non-small cell lung cancer	Durvalumab (MEDI4736)/Monalizumab/ Oleclumab (MEDI9447)/AZD6738	II	Not yet recruiting	120	Assistance Publique Hopitaux De Marseille
	NCT02331875	Squamous cell carcinoma of the oral cavity	IPH2201	I/II	Terminated	3	Innate Pharma
	NCT03088059	Squamous cell carcinoma of head and neck	Afatinib/Palbociclib/ IPH2201/Durvalumab/ Niraparib/BAY1163877	II	Recruiting	340	European Organization for Research and Treatment of Cancer

## Killer Cell Lectin-Like Receptor Family

### NKG2A

NK group 2 member A (NKG2A) is a type II membrane receptor that forms a heterodimer with CD94 (23). NKG2A binds a human leukocyte antigen (HLA) class I molecule (HLA-E) (24) and transduces inhibitory signaling that suppresses the cytokine secretion and cytotoxicity of NK cells (25–27). NKG2A^+^ NK cells infiltrate the tumor microenvironment, and increased expression of NKG2A in NK cells has been observed in patients with non-small cell lung cancer (28, 29), breast cancer (30), colorectal cancer (31, 32), acute myeloid leukemia (33, 34), hepatocellular carcinoma (35), breast cancer (36), cervical cancer (32), etc. A large proportion of NK cells with high NKG2A expression has also been found in tumor-draining lymph nodes (36). NK cells with elevated NKG2A expression are functionally exhausted and associated with a poor prognosis in human hepatocellular carcinoma (35). NKG2A expression predominantly increases on CD56^dim^ NK cells compared to CD56^bright^ NK cells, and these NKG2A^+^ CD56^dim^ NK cells are functionally exhausted and highly correlated with massive tumor size in human hepatocellular carcinoma (35).

Due to the strong capability of NKG2A to suppress NK cells, blockade of NKG2A is effective in restoring functions of NK cells. A mAb targeting NKG2A, namely, monalizumab (formerly IPH2201), has been tested in both phase I and phase II clinical trials (Table 2). NKG2A is overexpressed in NK cells from chronic lymphocytic leukemia patients, and blocking NKG2A with monalizumab is sufficient to restore the direct cytotoxicity of NK cells against HLA-E-expressing tumor cells (37). Treatment with IPH2201 has been shown to trigger NKG2A^+^ NK cell-mediated lysis of HLA-E^+^ target cells *in vitro* and abolish HLA-E^+^ leukemia and lymphoma tumors in xenograft mouse models of human neoplastic disease (NOD-SCID mice injected with HLA-E^+^ Epstein-Barr virus-positive cells or acute myeloid leukemia cells) (38). Interestingly, although NKG2A is predominantly expressed by NK cells, a study by the Vivier group showed that blockade of NKG2A enhanced the effector functions of both NK cells and CD8^+^ T cells in mice and humans (32). The use of monalizumab not only promoted human NK cell antibody-dependent cell-mediated cytotoxicity (ADCC) against various tumor cells but also rescued the function of CD8^+^ T cells when combined with PD-1 blockade (32). This group also reported impressive clinical outcomes: the use of monalizumab combined with cetuximab (an anti-EGFR antibody) in previously treated patients with squamous cell carcinoma of the head and neck showed a 30% response rate with limited side effects [fatigue (17%), pyrexia (13%), and headache (10%)] (32). Interestingly, a study by Kamiya et al. showed that NKG2A^null^ NK cells, which were generated through transduction of anti-NKG2A protein expression blockers (PEBLs), exhibited relatively high cytotoxicity against HLA-E^+^ tumor cells; moreover, this method generated more potent cytotoxicity than blockade with an anti-NKG2A mAb (39), suggesting a new method for developing NKG2A-targeted cancer immunotherapy.

## Killer Cell Immunoglobulin-Like Receptor Family

### KIRs

The killer-cell immunoglobulin-like receptors (KIRs) on human NK cells include both activating and inhibitory receptors, among which the inhibitory KIRs exhibit an inhibitory signaling motif and are named with the convention KIRxDL (40). KIR2DL and KIR3DL specifically bind to HLA-C and HLA-A/B allotypes, respectively (41, 42). KIR2DL includes KIR2DL1 and KIR2DL2/3, which bind distinct HLA-C allotypes to suppress the activation and effector functions of NK cells (41). Tumor cells induce the upregulated expression of KIRs on NK cells; for example, the expression of KIR2DL2 and HLA-C1 is significantly elevated in breast cancer patients (43); KIR2D (L1, L3, L4, and S4) and KIR3DL1 are expressed on tumor cells and TILs from non-small cell lung cancer patients, and patients without expression of KIR2D (L1, L3, L4, and S4) or KIR3DL1 on their tumor cells or TILs exhibit extended overall survival (44). KIR centromeric B haplotype is associated with significant risks of multiple basal cell carcinoma and squamous cell carcinoma, suggesting that interactions between KIRs and HLA molecules may modify the risks of basal cell carcinoma and squamous cell carcinoma (45). Interestingly, patients with bile duct cancer show multiple alterations at KIR gene loci (46), and genetic variations in KIRs are also present in non-small cell lung cancer patients who are resistant to anti-PD-1 monotherapy (47).

Due to their impressive suppressive effect on NK cells, human mAbs targeting KIRs have shown some clinical benefits. Lirilumab (1-7F9, IPH2101) targeting KIR2DL1, KIR2DL2, and KIR2DL3 increases NK cell cytotoxicity against autologous acute myeloid leukemia blasts and mediates the lysis of HLA-C-expressing tumor cells both *in vitro* and *in vivo* (48). Lirilumab also enhances NK cell activity against autologous multiple myeloma cells by preventing inhibitory KIR-ligand interactions (49). Phase I studies of lirilumab in patients with acute myeloid leukemia, hematological malignancies or solid tumors have shown that lirilumab can effectively block KIRs with mild adverse events (50, 51). However, a study by Carlsten et al. demonstrated that lirilumab not only reduced KIR2D expression on NK cells but also rapidly reduced NK cell functions, resulting in significantly diminished overall responses (52). On the other hand, IPH4102 targeting KIR3DL2 shows encouraging clinical activity in patients with relapsed or refractory cutaneous T-cell lymphoma, particularly those with Sézary syndrome (53).

An *in vitro* study found that stimulation with IL-12/IL-15/IL-18 also downregulated the expression of KIR2DL2/3, KIR2DL1, and KIR3DL1 on peripheral blood NK cells, resulting in reduced inhibitory KIR signaling and elevated CD16-dependent cytotoxicity (54). Furthermore, these IL-12/IL-15/IL-18-stimulated NK cells showed increased cytotoxicity against tumor cells (54).

## Immunoglobulin Superfamily

### TIGIT

TIGIT is an immunoglobulin protein that belongs to the CD28 family (55, 56). It was discovered as a surface receptor on T cells that recognizes CD155 in 2009 (57); however, TIGIT is also expressed on NK cells and interacts with other ligands, such as CD112 and CD113 (56). Together with CD226 and CD96, TIGIT participates in a balanced system to control the activation and function of T cells and NK cells. Unlike CD96, which inhibits only IFN-γ production in NK cells and has no effects on cytotoxicity, TIGIT can inhibit NK cell cytotoxicity directly through its ITIM domain in both humans and mice (58, 59). A study showed that the cytotoxicity of YTS NK cells (human NK cell line) transfected with TIGIT was strongly inhibited by CD155-transfected 721.221 cells and this inhibition could be blocked with an anti-TIGIT mAb *in vitro* (58). Furthermore, blockade of TIGIT has also been shown to significantly increase mouse NK cell-mediated killing of CD155-expressing B12 cells and enhance the secretion of IFN-γ (59). In an *in vivo* study, NK cells isolated from TIGIT-transgenic mice produced reduced amounts of IFN-γ after incubation with Yac-1 cells (murine T cell lymphoma cell line), while NK cells isolated from TIGIT^−/−^ mice produced increased amounts of IFN-γ. The suppression of IFN-γ production was mediated by TIGIT-CD155 ligation through the NF-κB pathway (60). In humans, NK cells with lower levels of TIGIT isolated from healthy individuals were shown to have a higher ability to secrete cytokines, degranulate, and kill target cells than those with higher TIGIT expression (61), suggesting the ability of TIGIT to regulate immune responses.

The expression of TIGIT is highly variable among different cancer types. The highest expressions of TIGIT in lymphocytes are found in Hodgkin's lymphoma, Warthin's tumors, medullary breast cancer, intestinal stomach cancer, and seminoma, while the lowest expressions of TIGIT in lymphocytes are found in renal oncocytoma, papillary renal cell cancer, desmoid tumors, pancreatic neuroendocrine cancer, chromophobic renal cell cancer, and adrenocortical cancer (62). Indeed, TIGIT^−/−^ mice show no resistance to lung metastasis in three different experimental lung metastasis models (B16F10, murine melanoma cell line; RM-1, murine prostate cancer cell line; E0771, murine breast cancer cell line) (63); moreover, TIGIT expression on NK cells is not significantly different between pancreatic cancer patients and healthy controls (64). Furthermore, a reduced proportion of TIGIT^+^ NK cells has been observed in the intratumoral region of hepatocellular carcinoma compared to the peritumoral region (65).

In contrast, TIGIT is overexpressed on CD8^+^ tumor-infiltrating lymphocytes (TILs) and tumor antigen-specific CD8^+^ T cells from melanoma patients and often coexpressed with the inhibitory receptor PD-1 (66). Coblockade of TIGIT and PD-1 can reverse dysfunctions in CD8^+^ TILs and antigen-specific CD8^+^ T cells by increasing their proliferation and effector functions (66). TIGIT expression was also found to be significantly increased in CD4^+^ T cells from chronic lymphocytic leukemia patients, and an increased number of TIGIT^+^ CD4^+^ T cells was found in patients with advanced disease stage (67). Moreover, TIGIT^−/−^ was shown to significantly inhibit tumorigenicity in both CT26 tumor-bearing BALB/c mice and MC38 tumor-bearing C57BL/6 mice, whereas an anti-TIGIT mAb significantly inhibited tumor growth in both of these colorectal tumor models (68). Furthermore, a study showed that TIGIT^−/−^ mice intravenously injected with B16 melanoma cells had relatively few lung metastases and improved overall survival (13). It is also important to note that TIGIT inhibits IFN-γ secretion of both CD8^+^ T cells and NK cells in the above mentioned colorectal tumor models; interestingly, CT26 tumor-bearing TIGIT knockout (KO) mice develop tumors early after NK cells are depleted, suggesting that NK cells and T cells collaborate to eliminate tumors (68).

The Tian group has demonstrated that TIGIT is highly expressed on tumor-infiltrating NK cells and associated with NK cell exhaustion in different tumor models [CT26 colon cancer, 4T1 breast cancer, B16 melanoma, and fibrosarcoma induced by methylcholanthrene (MCA)] and patients with colon cancer (13). NK cell-specific TIGIT KO in mice results in significantly prolonged survival, while TIGIT blockade inhibits NK cell exhaustion in colon tumors, breast tumors, and MCA-induced fibrosarcomas (13). Surprisingly, they showed that anti-TIGIT mAb could reduce tumor mass and slow tumor growth in T cell-deficient mice; in addition, NK cell deficiency resulted in an increased metastasis and number of exhausted CD8^+^ T cells, and abolished the effect of TIGIT blockade even in the presence of TIGIT-expressing CD8^+^ T cells (13). Notably, the therapeutic effects of anti-TIGIT mAbs, anti-PD-L1 mAbs or anti-TIGIT mAbs combined with anti-PD-L1 mAbs all depended on the presence of NK cells (13), indicating the importance of NK cells in checkpoint-targeted immunotherapy. Other studies have also indicated the importance of TIGIT^+^ NK cells in the tumor microenvironment. For example, blocking TIGIT could increase cytokine production by NK cells after an incubation with trastuzumab-coated breast cancer cells (69); the proportion of TIGIT^+^ NK cells was significantly increased in the peripheral blood mononuclear cell (PBMC) population of non-muscle invasive bladder cancer patients compared to that of healthy controls (70); endometrial tumor-resident CD103^+^ NK cells expressed higher levels of TIGIT than circulating CD103^−^ NK cells, and tumor-resident NK cells from patients with lymph node invasion showed significantly higher expressions of TIGIT than those from patients with no lymph node invasion (71); and TIGIT^+^ NK cells showed increased susceptibility to functional suppression by CD155-expressing myeloid-derived suppressor cells (MDSCs) (72). Currently, several clinical trials (phase I and phase II) focused on testing the feasibility of targeting this new pathway and improving therapeutic effects through combination with existing immunotherapies are either active or recruiting (Table 2).

### CD96

CD96 is a transmembrane glycoprotein that belongs to the immunoglobulin superfamily (73, 74). It was identified as a key receptor on NK cells that recognizes the ligand CD155 in 2004 (75) and was initially identified as a possible costimulatory receptor. However, 10 years later, its inhibitory characteristics were revealed by the Smyth group (76). Their study showed that CD96 competes with CD226 for CD155 binding and negatively regulates IFN-γ secretion in NK cells (77); however, it does not affect the direct killing of tumor cells by NK cells. Furthermore, CD96^−/−^ mice are resistant to MCA-induced fibrosarcoma and experimental lung metastasis modeled by injecting B16F10 melanoma cells (77), and blocking CD96 with a mAb inhibits experimental metastases in three different models (B16F10 melanoma, 3LL lung carcinoma, and RM-1 prostate cancer) (63). Blockade of the CD96-CD155 interaction was also shown to be effective in controlling lung metastases in NCR2-transgenic mice injected with B16-PDGFD cells (78). An anti-CD96 mAb was shown to be superior to other well-characterized checkpoint inhibitors, such as anti-CTLA-4 and anti-PD-1 antibodies, and the combination of an anti-CD96 mAb with an anti-CTLA-4 or anti-PD-1/PD-L1 mAb could further inhibit experimental lung metastases (63). Notably, although CD96 was also expressed by T cells, the control of metastases by an anti-CD96 mAb appeared to be dependent on NK cells, CD226 and IFN-γ production (63), suggesting a non-negligible role for NK cells in cancer immunotherapy. Further, blocking CD96 reduced the number of B16F10 metastases in Tigit^−/−^ mice compared to wildtype mice, indicating the synergistic potential of blocking CD96 and TIGIT in treating cancer (63).

The structural basis for the CD96-CD155 interaction involves the “ancillary key” motif that is critical for CD155 recognition; moreover, CD96 and CD155 interact via the “lock-and-key” docking mode (79). However, surprisingly, a comparison between three anti-CD96 mAbs, including two that block the CD96-CD155 interaction (3.3 and 6A6) and one that does not block this interaction (8B10), revealed that although the two blocking mAbs showed higher potency than the non-blocking mAb in the control of metastases, it was not necessary to block the CD96-CD155 interaction to promote NK cell antimetastatic functions (80, 81). In contrast, another study using a transgenic mouse model of resectable pancreatic ductal adenocarcinoma showed that a mAb targeting the CD96-CD155 interaction (6A6) significantly reduced distant metastases, while a mAb that did not target the CD96-CD155 interaction (8B10) showed no effect on the frequency of metastases (82). One possible explanation for the contradictions occurred involves various microenvironmental cues in *in vitro* vs. *in vivo* settings, given that the microenvironment in an *in vivo* experiment is much more complicated and involves various cell-cell interactions and consequences (for example, cytokine secretions, etc.) following these interactions, which may contribute to the differences raised between the studies.

In humans, a significantly decreased percentage of CD96^+^ NK cells in pancreatic cancer patients and associations of this decreased percentage with lymph node metastasis and tumor histological grade were observed (64), suggesting a possible protective role for CD96^+^ NK cells in pancreatic cancer. Contradictorily, another study showed increased serum levels of soluble CD96 in NK cells from late-stage melanoma patients (83). In addition, a study noted an elevated proportion and number of CD96^+^CD56^dim^ NK cells in hepatocellular carcinoma tissues, and these NK cells were functionally exhausted with impaired IFN-γ and TNF-α productions (65). Furthermore, patients with higher CD96^+^ NK cell infiltration within tumors have been shown to exhibit relatively short disease-free survival times (65). These studies suggest a protumor role for CD96^+^ NK cells in melanoma and hepatocellular carcinoma.

### LAG-3

LAG-3 is a negative coinhibitory receptor expressed on T cells and NK cells that binds MHC class II (MHC-II) molecules on target cells (84, 85). LAG-3 also interacts with LSECtin, a cell surface lectin that belongs to the C-type lectin receptor superfamily, to inhibit IFN-γ production by effector T cells (86). Recently, Chen and colleagues identified fibrinogen-like protein 1 (FGL1), a liver-secreted protein, as an MHC-II-independent ligand for LAG-3 in both humans and mice (87). Previous studies have shown that LAG-3 negatively regulates the proliferation and activation of T cells (88, 89) and that it also interacts with FGL1 to inhibit antigen-mediated T cell responses both *in vitro* and *in vivo* (87). LAG-3 and PD-L1 coregulate the exhaustion of CD8^+^ T cells, and compared to anti-PD-L1 mAb or anti-LAG-3 mAb monotherapy, dual blockade of LAG-3 and PD-L1 increases the number and effector functions of functional virus-specific CD8^+^ T cells (90), suggesting that the combination of anti-LAG-3 and anti-PD-L1 antibodies results in an improved reversal of exhaustion.

LAG-3 has been shown to suppress immune responses in several tumors, including Hodgkin's lymphoma, gastric cancer, breast cancer, and other solid tumors (91). In studies of squamous cell carcinoma mouse models, both CD8^+^ and CD4^+^ TILs coexpressed LAG-3 and PD-1, and dual blockade of LAG-3 and PD-1 significantly suppressed tumor growth (92). LAG-3 has been detected in TILs from 41.5% of non-small cell lung cancer patients and associated with the checkpoint molecules PD-1 and TIM-3 (93). Moreover, elevated LAG-3 expression has been associated with reduced progression-free survival in patients with advanced non-small cell lung cancer treated with PD-1 blockade (93). Elevated expression of LAG-3 has also been observed in patients with peripheral T cell lymphoma or NK/T cell lymphoma (94). LAG-3^+^ TIL numbers are increased in MHC-II^+^ tumors (lung cancer, melanoma, and breast cancer), and MHC-II^+^ tumors acquire immunosuppressive signals through LAG-3; thus, combined PD-1/PD-L1 and LAG-3 blockade can provide a particular advantage against MHC-II^+^ tumors (95). Interestingly, expression of the newly identified ligand FGL1 has also been shown to be upregulated in cancer, and blockade of the FGL1-LAG-3 interaction stimulates immune responses and exhibits therapeutic effects on mouse tumor models (MC38 colon cancer and Hepa1-6 liver cancer) (87). Furthermore, compared to monotherapy, an anti-FGL1 mAb or anti-LAG-3 mAb in combination with an anti-B7-H1 mAb significantly reduces tumor burden and prolongs survival (87).

An early study in 1996 indicated that mice lacking the *lag3* gene exhibited reduced lysis of Yac-1 cells; in addition, polyclonal antibodies against LAG-3 could reduce NK-mediated lysis of Yac-1 cells but leave MHC-II-deficient target cells intact, suggesting the existence of an independent mode of natural killing through LAG-3 (96). However, this independent mode of natural killing has not been observed with human NK cells, and blockade of LAG-3 has no effect on the natural killing of various target cells (97). Notably, although studies on LAG-3^+^ NK cells are limited, the role of these cells in antitumor immunity should not be neglected. A study showed that using IL-12 to boost the cytotoxicity of NK cells in a lung cancer model (BALB/c mice injected with 4T1 cells) increased the NK cell population expressing high levels of coinhibitory molecules, including LAG-3, which limited NK cell-mediated antimetastatic activity (98). The combination of an anti-LAG-3 mAb with IL-12 significantly reduces lung metastasis, whereas monotherapy fails to achieve this effect (98). Furthermore, synergy between an anti-LAG-3 mAb and IL-12 contributes to the increased efficacy of IL-12 immunotherapy in breast cancer, which is solely dependent on NK cells, suggesting that LAG-3 is applicable in not only T cell-mediated immunotherapies but also NK cell-mediated antimetastatic immunotherapies (98).

### TIM-3

TIM-3 is a type I glycoprotein that binds galectin-9 (Gal-9), high mobility group box 1 protein (HMGB1), and carcinoembryonic antigen-related cell adhesion molecule 1 (CEACAM-1) on target cells to act as an NK cell coreceptor (91, 99, 100). An early *in vitro* study showed that an NK92 cell line overexpressing TIM-3 secreted an increased amount of IFN-γ, while TIM-3 blockade resulted in reduced IFN-γ production (101). However, although human TIM-3^+^ NK cells are functional in terms of cytokine production and cytotoxicity, they become suppressed when TIM-3 is cross-linked with antibodies (102), suggesting that an interaction between TIM-3 and its ligand can result in NK cell dysfunction.

TIM-3 can mediate cell exhaustion and suppress immune responses under both chronic viral and cancerous conditions. For example, TIM-3 mediates suppression of NK cells in chronic hepatitis B patients, while TIM-3 blockade results in increased NK cell cytotoxicity both *in vitro* and *ex vivo* (103). TIM-3 is highly expressed in various tumor types, including gastrointestinal stromal tumor (104), lung adenocarcinoma (105), perineural squamous cell carcinoma (106), melanoma (107), gastric cancer (108), acute myeloid leukemia (109), colon cancer (110), bladder cancer (70), renal cell carcinoma (111), pancreatic cancer (112), glioma (113), anaplastic thyroid cancer (114), peripheral T cell lymphoma, NK/T cell lymphoma (94), etc. Significant overexpression of TIM-3 has been observed in peripheral NK cells from non-muscle invasive bladder cancer patients (70); moreover, TIM-3 is expressed in TILs from 25.3% of non-small cell lung cancer patients and associated with the expression of PD-1 and LAG-3 (93). TIM-3 expression was found to be higher on peripheral NK cells from glioma patients than on those from healthy controls, and these TIM-3^+^ NK cells showed a reduced capability for IFN-γ production and correlated with the proportion of Ki-67^+^ tumor cells (113). Furthermore, TIM-3 expression is upregulated on NK cells in late-stage melanoma patients, and blockade of TIM-3 reverses NK cell exhaustion in these patients (107).

TIM-3 functions as a potential prognostic marker in several tumor types. Upregulated expression of TIM-3 in peripheral NK cells from lung adenocarcinoma patients correlates with decreased overall survival, while blockade of TIM-3 enhances cytotoxicity and IFN-γ production in peripheral NK cells (105). Overexpression of TIM-3 in NK cells from gastric cancer patients has been associated with an advanced tumor stage (108). In addition, a study found that endometrial tumor-resident CD103^+^ NK cells expressed higher levels of TIM-3 than circulating CD103^−^ NK cells, and tumor NK cells from patients with lymph node invasion showed significantly higher expression of TIM-3 than those from patients with no lymph node invasion (71). Bladder cancer patients have high levels of TIM-3^+^ NK cells and Gal-9^+^ tumor cells, and patients with relatively low levels of TIM-3^+^ NK cells and Gal-9^+^ tumor cells have an improved prognosis (115). Furthermore, TIM-3^+^ NK cells are defective in esophageal cancer, and relatively high TIM-3 expression on NK cells correlates with a poor prognosis in esophageal carcinoma (116).

However, contradictorily, studies have also reported stimulatory functions of TIM-3 (117). For example, after short-term stimulation with anti-CD3/CD28 antibodies, TIM-3 can enhance the secretion of IL-2 and signaling pathways that lead to T-cell activation (118, 119). TIM-3 engagement during antigen stimulation directly promoted CD8 T cell differentiation through mTORC1 (120). Furthermore, activation of human T cells was not affected by the presence of Gal-9 or antibodies to TIM-3 (121), which also reported a contradictory role of Gal-9 as a ligand for TIM-3. These studies suggest that the use of anti-TIM-3 should be particularly careful because TIM-3 also plays an activating role under certain circumstances, whether its antibodies act as agonist or antagonist remains to be questioned. Both anti-murine and anti-human TIM-3 antibodies bind to TIM-3 in a manner that interfere with the binding of TIM-3 to both phosphatidylserine and CEACAM1, the understanding of the interaction between TIM-3 and its ligands plays an important role in the screening of anti-TIM-3 antibody candidates (122).

Some studies have explored the reasons underlying the upregulation of TIM-3 expression on NK cells in the tumor microenvironment. One study reported that the LPHN1/PKC/mTOR-TIM-3-Gal-9 pathway in human acute myeloid leukemia induced high levels of Gal-9 secretion and the release of soluble TIM-3 (109). Gal-9 impaired the killing of tumor cells by NK cells, whereas soluble TIM-3 impaired the ability of T cells to produce IL-2, contributing to the breakdown of immune surveillance and thus to the progression of tumors (109). Another proposed mechanism is that MHC-I-deficient tumors induce coexpression of TIM-3 and PD-1 on NK cells, resulting in functional NK cell exhaustion in both tumor-bearing mice and cancer patients; functional recovery in these exhausted NK cells induced by vaccination requires IL-21 produced by NKT cells (110). Furthermore, carcinoma-associated fibroblasts (CAFs) have been shown to promote the expression of TIM-3 in pancreatic cancer patients (112). Sustained IL-15 stimulation upregulates TIM-3 expression on both T and NK cells (123). TIM-3 expression can also be induced by TNF-α through the NF-κB signaling pathway (116). In addition, IL-27/NFIL3 signaling axis has been identified crucial for the induction of Tim-3, IL-10 and T-cell dysfunction (124).

## Perspective

The success of mAbs targeting CTLA-4 and PD-1 has shed light on cancer immunotherapy, and restoring exhausted T cells has shown promising clinical outcomes in some patients. However, there were still many patients who are nonresponsive to these treatments. However, recent findings indicate that improved survival highly correlates with the frequency of DNAM-1^+^CD56^dim^ NK and NKp46^+^CD56^dim^ NK cells after treatment with anti-CTLA-4 in patients with malignant mesothelioma (125). Hsu and Hodgins et al. demonstrated in multiple tumor models that PD-1 is upregulated on the most activated and functionally responsive intratumoral NK cells, suggesting that the efficacy of PD-1 blockade depends in part on inducing an NK cell-based antitumor response (126). Thereof, we propose that NK cell-targeted immunotherapy may provide an alternative or complementary approach to overcome the limitations of T cell immunotherapy and combination with NK cell immunotherapy could increase the response rate of T cell-targeted treatments. NK cells are critical for immunosurveillance, particularly in the control of metastasis and hematological cancers. A study by the Tian group indicated the importance of NK cells in checkpoint immunotherapy; TIGIT blockade prevented NK cell exhaustion in the absence of T cells and B cells, and an anti-TIGIT mAb improved T cell responses in an NK cell-dependent manner (13). These findings suggest that certain checkpoint molecules expressed by both T cells and NK cells may exert a greater effect on NK cells than on T cells and that NK cells could be essential for the T cell-mediated antitumor response in such a scenario.

The recent success of anti-NKG2A mAb on clinical trials unleashes its role as a promising checkpoint inhibitor in treating cancers with minimum side effects, and the success of NKG2A blockade also points out the importance of NK cells in anti-tumor immunity and advances the idea that combined reversal of both T and NK cell exhaustion is truly important in anti-tumor immunotherapy. Anti-NKG2A could be the third potential checkpoint inhibitor approved by the FDA following anti-CTLA-4 and anti-PD-1/PD-L1. Another promising checkpoint molecule targeting NK cells is TIGIT based on the results observed by the Tian group (13). However, due to its constitutive expression on peripheral human NK cells, more studies are required to fulfill its role as a checkpoint in treating cancer. The balance between CD96, TIGIT, and CD226 is critical for proper immune responses by NK cells. The accumulation of CD96 in NK cells in hepatocellular carcinoma patients disrupts the balance between these three receptors, which subsequently results in NK cell dysfunction and exhaustion (65); therefore, careful examination of the CD96-TIGIT-CD226 system should be involved in developing immunotherapies targeting these receptors. In addition to CD155, CD112, and CD113, human TIGIT can also bind to the Fap2 protein of *Fusobacterium nucleatum*, and the interaction between TIGIT and Fap2 inhibits NK cell cytotoxicity (127). This finding unleashes a new mechanism of tumor immune evasion that depends on the bacterium and identifies new possibilities for NK cell immunotherapy. Alterations in KIR and HLA gene loci affect NK cell functions, which should be considered when developing immunotherapies against KIRs (46). The interaction between LAG-3 and the newly defined ligand FGL1 suppresses the functions of T cells, and whether FGL1 also interacts with LAG-3 on NK cells merits further research (87).

Other checkpoint molecules on NK cells have been proven to be potential targets, and further experiments are needed to prove these novel targets for NK-based immunotherapy (22, 128). There are ways to improve the efficacy of NK cell immunotherapy. For example, NKG2A^null^ NK cells are more effective than NKG2A^+^ NK cells treated with an anti-NKG2A mAb, suggesting a new immunotherapeutic approach using NKG2A^null^ NK cells (39, 129). In addition, cytokines can enhance the efficacy of mAbs (130), and a combination of cytokine treatment with checkpoint immunotherapy may boost the effects of mAbs. Furthermore, we believe that combined blockade of checkpoint molecules expressed by T cells and NK cells could unleash antitumor immunity mediated by innate and adaptive populations, which not only improve overall antitumor immune responses but also allow the two approaches to complement each other; this strategy might be the solution for the “non-responders.” Accumulating evidence suggests that NK cell-targeted immunotherapy is highly feasible; however, our knowledge of the inhibitory mechanisms in NK cells is still inadequate, and more fundamental research is required to identify the best inhibitory pathways to be targeted for future clinical applications.

## Author Contributions

HS and CS were involved in the search and analysis of the literature, design and writing of the manuscript, and revision of the manuscript.

### Conflict of Interest

The authors declare that the research was conducted in the absence of any commercial or financial relationships that could be construed as a potential conflict of interest.

## Abbreviations

NK: Natural killer
CTL: Cytotoxic T lymphocyte
PBMC: Peripheral blood mononuclear cell
MDSC: Myeloid-derived suppressor cell
mAb: Monoclonal antibody
IFN: Interferon
TNF: Tumor necrosis factor
IL: Interleukin
KO: Knockout
HLA: Human leukocyte antigen
ADCC: Antibody-dependent cell-mediated cytotoxicity
MHC: Major histocompatibility complex
FDA: Food and Drug Administration
MCA: Methylcholanthrene
PD-1: Programmed death-1
CTLA-4: Cytotoxic T-lymphocyte-associated protein 4
TIM-3: T cell immunoglobulin and mucin domain 3
TIGIT: T cell immunoreceptor with Ig and ITIM domains
KIR: Killer cell immunoglobulin-like receptor
LAG-3: Lymphocyte activating gene 3
FGL1: Fibrinogen-like protein 1
NKG2A: NK group 2 member A
Gal-9: Galectin-9
CAF: Carcinoma-associated fibroblast
PEBL: Protein expression blocker
HMGB1: High mobility group box 1 protein
CEACAM-1: Carcinoembryonic antigen-related cell adhesion molecule 1.
